# First-Time-in-Human Study and Prediction of Early Bactericidal Activity for GSK3036656, a Potent Leucyl-tRNA Synthetase Inhibitor for Tuberculosis Treatment

**DOI:** 10.1128/AAC.00240-19

**Published:** 2019-07-25

**Authors:** David Tenero, Geo Derimanov, Alex Carlton, John Tonkyn, Matt Davies, Simon Cozens, Stephanie Gresham, Alison Gaudion, Adeep Puri, Morris Muliaditan, Joaquin Rullas-Trincado, Alfonso Mendoza-Losana, Andrew Skingsley, David Barros-Aguirre

**Affiliations:** a GlaxoSmithKline Clinical Pharmacology Modeling and Simulation, Collegeville, Pennsylvania, USA; b GlaxoSmithKline Clinical Pharmacology and Experimental Medicine, Collegeville, Pennsylvania, USA; c GlaxoSmithKline Biostatistics, Stockley Park, London, United Kingdom; d GlaxoSmithKline Global Sciences and Delivery, Stevenage, Hertfordshire, United Kingdom; e GlaxoSmithKline Safety and Medical Governance, Stockley Park, London, United Kingdom; f GlaxoSmithKline, Translation Project Specialist Team, Ware, Hertfordshire, United Kingdom; g GlaxoSmithKline, Mechanistic Safety and Disposition, Ware, Hertfordshire, United Kingdom; h Hammersmith Medicines Research, London, United Kingdom; i GlaxoSmithKline DMPK Modelling, Stevenage, Hertfordshire, United Kingdom; j GlaxoSmithKline Global Health, TBDPU, Tres Cantos, Madrid, Spain; k GlaxoSmithKline Global Health Clinical Drug Development, Stockley Park, London, United Kingdom

**Keywords:** EBA prediction, FTIH, GSK3036656, clinical trial simulations, dose escalation, dose rationale, food effect, pharmacokinetics, repeat dose, safety, single dose, tolerability, tuberculosis

## Abstract

This first-time-in-human (FTIH) study evaluated the safety, tolerability, pharmacokinetics, and food effect of single and repeat oral doses of GSK3036656, a leucyl-tRNA synthetase inhibitor. In part A, GSK3036656 single doses of 5 mg (fed and fasted), 15 mg, and 25 mg and placebo were administered. In part B, repeat doses of 5 and 15 mg and placebo were administered for 14 days once daily. GSK3036656 showed dose-proportional increase following single-dose administration and after dosing for 14 days. The maximum concentration of drug in serum (*C*_max_) and area under the concentration-time curve from 0 h to the end of the dosing period (AUC_0–τ_) showed accumulation with repeated administration of approximately 2- to 3-fold. Pharmacokinetic parameters were not altered in the presence of food. Unchanged GSK3036656 was the only drug-related component detected in plasma and accounted for approximately 90% of drug-related material in urine. Based on total drug-related material detected in urine, the minimum absorbed doses after single (25 mg) and repeat (15 mg) dosing were 50 and 78%, respectively. Unchanged GSK3036656 represented at least 44% and 71% of the 25- and 15-mg doses, respectively. Clinical trial simulations were performed to guide dose escalation during the FTIH study and to predict the GSK3036656 dose range that produces the highest possible early bactericidal activity (EBA_0–14_) in the prospective phase II trial, with consideration of the predefined exposure limit. GSK3036656 was well tolerated after single and multiple doses, with no reports of serious adverse events. (This study has been registered at ClinicalTrials.gov under identifier NCT03075410.)

## Abstract (precis)

This first-time-in-human (FTIH) study evaluated the safety, tolerability, pharmacokinetics, and food effect of single and repeat oral doses of GSK3036656, a leucyl-tRNA synthetase inhibitor. In part A, GSK3036656 single doses of 5 mg (fed and fasted), 15 mg, and 25 mg and placebo were administered. In part B, repeat doses of 5 and 15 mg and placebo were administered for 14 days once daily.

## INTRODUCTION

Tuberculosis (TB), despite being a curable disease, remains a serious global public health threat. It is estimated that one-third of the world’s population is infected with Mycobacterium tuberculosis bacteria, leading to over 10 million new cases and nearly 1.6 million deaths alone in 2016 (1–6). Issues with current combination treatment regimens include length of treatment (6 to 18 months), significant side effects, emergence of drug resistance (7), and drug-drug interactions. Drugs recently approved for the treatment of TB by the U.S. Food and Drug Administration (FDA) and the European Medicines Agency (EMA) include bedaquiline and delamanid (8, 9); however, these drugs are currently licensed only for the treatment of drug-resistant TB, and their potential role in other treatment paradigms is not yet understood (10, 11). There is an urgent need to develop effective, affordable, accessible, and well-tolerated new therapies with novel modes of action to create regimens which can fill the gaps in current TB treatment. Over a dozen candidate drugs are under development for drug-susceptible, drug-resistant, and latent tuberculosis, including nine in early-phase trials (phases I and II) and three in advanced stages of development for multidrug-resistant TB (MDR-TB) (7).

GlaxoSmithKline (GSK) synthesized and preclinically evaluated a novel series of 3-aminomethyl 4-halogen benzoxaboroles as M. tuberculosis enzyme leucyl-tRNA synthetase (LeuRS) inhibitors. These compounds exhibited good anti-TB activity with high specificity and selectivity over the human mitochondrial and cytoplasmic LeuRS enzyme (12). From this series of compounds, GSK3036656 emerged as a promising lead candidate and was selected for further clinical development. GSK3036656 (molecular weight, 257.48 g/mol) has shown *in vitro* activity against M. tuberculosis bacterial strain H37Rv with an MIC value of 23.5 ng/ml and a 50% inhibitory concentration (IC_50_) value of 58.8 ng/ml in a LeuRS aminoacylation assay (12). It has exhibited promising activity against laboratory strains of M. tuberculosis as well against a selection of drug-sensitive TB (DS-TB), MDR-TB, and extensively drug-resistant TB (XDR-TB) clinical isolates. GSK3036656 has also shown specific selectivity for TB pathogens and inactivity against a panel of common bacterial pathogens as well as a panel of mammalian cell lines.

The efficacy of GSK3036656 was assessed in C57BL/6 mice having acute and chronic lung infections by M. tuberculosis H37Rv, which mimic infections by actively replicating and nonreplicating/slow-replicating mycobacteria, respectively (13, 14). In the acute model, 10 mice were administered different daily oral doses ranging from 0.1 to 100 mg/kg of body weight for 8 days starting 1 day after infection. A maximum difference of 3.6 log CFU was obtained in the lungs compared to untreated mice. These data were fitted to a sigmoidal curve, and the effective dose that gave the 90% of the total effect (ED_max_) was estimated as 1.1 mg/kg (IC_95_, 0.7 to 2.3 mg/kg). In the chronic model, a total of 18 mice were administered doses ranging from 0.1 to 30 mg/kg from weeks 6 to 14 after infection. Maximum reduction (drop of 2.1 log_10_ CFU in the lungs) was achieved after 2 months of daily oral treatment and after fitting the data to a sigmoidal curve. The ED_max_ for GSK3036656 was estimated as 1.3 mg/kg (IC_95_, 0.7 to 3.4 mg/kg). The plasma steady-state area under the concentration-time curve (AUC) associated with this ED_max_ (AUCED_max_) was 1,740 ng · h/ml and was considered to be the target exposure for efficacy in humans. Some additional groups treated at 0.3 to 30 mg/kg were studied in the chronic model to obtain kinetic data (from 2 to 16 weeks of treatment).

The principal findings observed in nonclinical toxicity studies following oral administration of GSK3036656 were cardiovascular and hematopoietic, with some minor changes in the liver, kidney, and thymus. The hematopoietic, kidney, liver, and thymus effects are considered either nonadverse or monitorable in the clinic. The structural cardiovascular changes that occurred in a single species at high doses were nonmonitorable but occurred at plasma concentrations where heart rate (HR) and blood pressure (BP) changes are recorded. Specific cardiovascular safety endpoints were included in the first-time-in-human (FTIH) study, including vital sign (HR and BP) stopping criteria. Additionally, a conservative fold margin from the human exposure (AUC and maximum concentration of drug in serum [*C*_max_]) to the NOAEL (AUC = 4,900 ng · h/ml; *C*_max_ = <443 ng/ml) was implemented.

GSK3036656 is a low-hepatic-clearance compound with high solubility and moderate passive permeability and is not a P-glycoprotein (P-gp) substrate, which therefore undergoes rapid absorption and exhibited very high oral bioavailability in the nonclinical species (mouse, rat, and dog) studied. *In vitro* data suggested that GSK3036656 plasma protein binding is low in the nonclinical species (20.8, 31.2, and 24.0% for mouse, rat, and dog, respectively) and humans (16.2%). Studies using hepatocytes showed metabolism in the nonclinical species via oxidation with deboronation. *In vitro* data showed minimal oxidative metabolism of GSK3036656, a lack of substrate specificity for P-gp or breast cancer resistance protein, and minimal evidence for direct inhibition of cytochrome P450s or OATP1B1, with no evidence for metabolism-dependent inhibition or induction of CYP3A4.

On account of the overall favorable nonclinical safety, efficacy, and pharmacokinetic (PK) profile, GSK3036656 was selected as a clinical candidate for further evaluation in healthy adult subjects. The objective of the FTIH study was to evaluate the safety, tolerability, PK, and food effect of single and repeat oral doses of GSK3036656. Pharmacokinetic results from this study were subsequently used in conjunction with an M. tuberculosis growth dynamics model and efficacy data from the mouse infection model to establish the dose range to be used in the subsequent phase II study.

## RESULTS

### Study conduct.

The study was monitored in accordance with ICH E6, section 5.18. There were no significant findings relating to noncompliance with good clinical practice (GCP) (e.g., potential serious misconduct, including potential serious breaches) identified during monitoring or auditing of a site. There were no important deviations related to study inclusion or exclusion criteria. The most common important protocol deviations across the study were related to out-of-window PK and safety assessments.

### Safety and tolerability.

In part A, overall, 20 subjects were screened, and 11 subjects were randomized. In the first dosing period, 9 subjects were randomized; however, during part A, 3 subjects withdrew (2 due to personal reasons and 1 due to reaction with electrocardiogram [ECG] tabs), and 2 were replaced. In part B, overall, 30 subjects were screened, and 19 subjects were randomized: 9 in the first cohort (7 receiving GSK3036656 at 5 mg and 2 receiving placebo) and 10 in the second cohort (8 receiving GSK3036656 at 15 mg and 2 receiving placebo). All subjects completed the study (5 mg [*n* = 7], 15 mg [*n* = 8], and placebo [*n* = 4]).

GSK3036656 was well tolerated, with no reports of serious adverse events (SAEs). Overall, 6 (55%) subjects reported AEs in part A, including 3 subjects who received GSK3036656 at 25 mg (Table 1). All AEs were reported to be mild. Musculoskeletal chest pain was reported in a 29-year-old male, lasting 90 min. It was nonserious, mild in intensity, and considered by the investigator to be unrelated to the study drug. The event occurred 7 days after dosing with GSK3036656 at 25 mg (the first dosing period for the subject). The investigator considered it to be exertion related, as the subject was known to exercise frequently. Medical device site dermatitis resolved on day 42 from its onset; all other AEs were resolved in 1 to 3 days from their onset.

**TABLE 1:** Overall summary of all adverse events by preferred term in part A (single dose)

Preferred term	No. (%) of subjects with AE in part A					
	Placebo (*n* = 9)^*a*^	GSK3036656, 5 mg (*n* = 6)	GSK3036656, 15 mg (*n* = 6)	GSK3036656, 25 mg (*n* = 6)	GSK3036656, 5 mg, fed (*n* = 5)	Total (n = 11)^*b*^
Any AE	1 (11)	1 (17)	1 (17)	3 (50)	1 (20)	6 (55)
Abdominal pain	0	0	0	2 (33)^*c*^	0	2 (18)
Headache	0	0	1 (17)	1 (17)	0	2 (18)
Cough	0	0	0	0	1 (20)	1 (9)
Dyspepsia	1 (11)	0	0	0	0	1 (9)
Epistaxis	0	1 (17)	0	0	0	1 (9)
Medical device site dermatitis	0	0	0	1 (17)	0	1 (9)
Musculoskeletal chest pain	0	0	0	1 (17)	0	1 (9)

a The placebo group includes all placebo doses taken throughout study part A, including subjects who received placebo twice.

b Includes the 9 subjects originally randomized and the two replacement subjects.

c Abdominal pain in one subject was judged to be possibly drug related.

In part B, 2 subjects in the 5-mg repeat-dose GSK3036656 group and 1 subject in the 15-mg repeat-dose GSK3036656 group reported an AE; all AEs were reported to be mild or moderate. The most frequently reported AE was headache (two subjects) (Table 2). All other AEs were reported in 1 subject each (dizziness, diarrhea, medical device site reaction, infectious mononucleosis, and increased liver function test values). All of the AEs except diarrhea were reported in the 5-mg repeat-dose GSK3036656 group. One subject in the 5-mg group was diagnosed with acute infectious mononucleosis based on clinical signs and symptoms (severe pharyngitis with flu-like symptoms) and supportive laboratory findings (antibodies to Epstein-Barr viral capsid antigen positive for IgM and indeterminate for IgG). This AE was judged to be unrelated to the study drug. This subject also experienced AEs of headache, dizziness, and raised liver enzyme levels, all attributed to acute infectious mononucleosis. Three subjects from the 15-mg repeat-daily-dosing group exceeded the predefined AUC from 0 to 24 h (AUC_0–24_) safety exposure limit. No AEs were reported in these subjects. None of the abnormal vital signs or laboratory findings (clinical chemistry, hematology, and urine analysis) reported during both parts of the study were judged to be AEs (except for the liver function tests in the subject with infectious mononucleosis). All abnormal ECG findings during the study were considered not clinically significant by the investigator. All telemetry evaluations in both parts of the study were judged to be normal.

**TABLE 2:** Overall summary of adverse events by preferred term in part B (repeat dose)^*a*^

Preferred term	No. (%) of subjects with AE in part B		
	Placebo (*n* = 4)	GSK3036656, 5 mg (*n* = 7)	GSK3036656, 15 mg (*n* = 8)
Any AE	0	2 (29)	1 (13)
Headache	0	2 (29)	0
Dizziness	0	1 (14)	0
Diarrhea	0	0	1 (13)
Medical device site reaction	0	1 (14)	0
Infectious mononucleosis	0	1 (14)	0
Increased liver function test	0	1 (14)	0

a Note that none of the adverse events were judged to be possibly drug related.

### Pharmacokinetics and metabolism.

**FIG 1:**
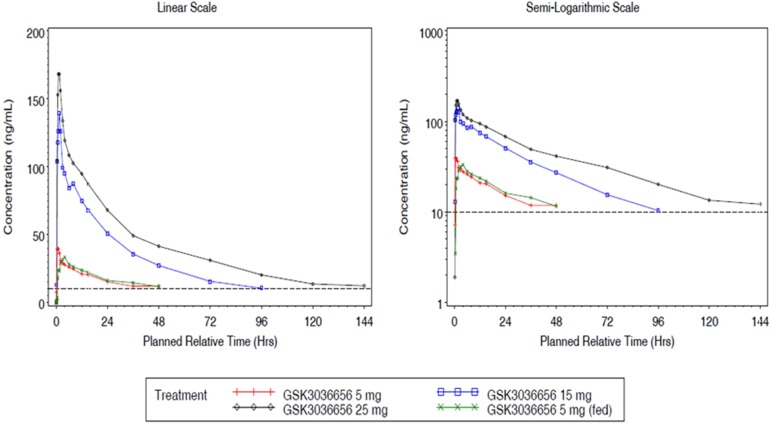
Mean plasma GSK3036656 concentration-time plots (linear and semilog) for part A. Note that the lower limit of quantification (LLQ) is 10 ng/ml.

**FIG 2:**
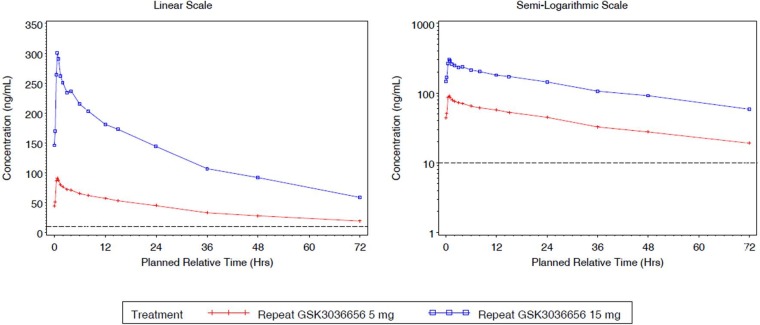
Mean plasma GSK3036656 concentration-time plots (linear and semilog) for part B (day 14). Note that the LLQ is 10 ng/ml.

GSK3036656 single-dose and repeat-dose pharmacokinetic parameters are shown in Tables 3 and 4. Mean GSK3036656 single-dose and repeat-dose concentration-time profiles are shown in Fig. 1 and 2. Following single-dose oral administration of GSK3036656 at 5, 15, and 25 mg in part A, both *C*_max_ and AUC extrapolated to infinity (AUC_0–∞_) increased in a dose-proportional manner (slope of 0.96).

**TABLE 3:** Plasma pharmacokinetic parameters of GSK3036656 after single-dose administration^*a*^

Treatment dose (mg) for part A	Geometric mean AUC_0–∞_ (h · ng/ml) (%CVb)	Geometric mean AUC_0–_*_t_* (h · ng/ml) (%CVb)	Geometric mean *C*_max_ (ng/ml) (%CVb)	Mean *t*_1/2_ (h) (SD)	Mean *T*_max_ (h) (SD)
5 (*n* = 6)	1,404.5 (29.9)	771.0 (26.0)	49.06 (30.5)	40.9 (16.1)	1.1 (0.5)
15 (*n* = 6)	3,796.1 (19.8)	3,252.9 (24.1)	177.61 (23.1)	28.4 (3.0)	1.1 (0.6)
25 (*n* = 6)	6,557.8 (13.8)	5,686.4 (11.4)	207.38 (18.5)	49.9 (13.1)	1.1 (0.5)
5 (fed) (*n* = 5)	1,450.7 (9.4)	782.8 (18.1)	47.37 (23.5)	34.8 (6.6)	2.2 (1.3)

a %CVb, percent coefficient of variance between subjects.

**TABLE 4:** Plasma pharmacokinetic parameters of GSK3036656 after repeat-dose administration

Parameter^*a*^	Treatment regimen (part B)	Visit	No. of subjects	Value
Geometric mean AUC_0–_*_t_* (h · ng/ml) (%CVb)	Repeat 5 mg	Period 1 day 1	7	485.6 (12.3)
	Repeat 15 mg	Period 2 day 1	8	1,891.0 (9.9)

Geometric mean AUC_0–τ_ (h · ng/ml) (%CVb)	Repeat 5 mg	Period 1 day 14	7	1,392.2 (12.5)
	Repeat 15 mg	Period 2 day 14	8	4,461.3 (23.7)

Geometric mean *C*_max_ (ng/ml) (%CVb)	Repeat 5 mg	Period 1 day 1	7	48.4 (33.6)
		Period 1 day 14	7	97.0 (19.8)
	Repeat 15 mg	Period 2 day 1	8	178.8 (32.8)
		Period 2 day 14	8	309.6 (20.2)

Geometric mean *R_o_*^*b*^ (%CVb)	Repeat 5 mg	Period 1 day 14	7	2.9 (9.1)
	Repeat 15 mg	Period 2 day 14	8	2.4 (21.6)

Geometric mean *R_o_C*_max_^*c*^ (%CV)	Repeat 5 mg	Period 1 day 14	7	2.0 (24.3)
	Repeat 15 mg	Period 2 day 14	8	1.7 (26.7)

Mean *t*_1/2_ (h) (SD)	Repeat 5 mg	Period 1 day 14	7	44.0 (11.4)
	Repeat 15 mg	Period 2 day 14	8	37.0 (9.2)

Mean *T*_max_ (h) (SD)	Repeat 5 mg	Period 1 day 1	7	1.3 (0.9)
		Period 1 day 14	7	0.8 (0.3)
	Repeat 15 mg	Period 2 day 1	8	0.8 (0.3)
		Period 2 day 14	8	0.8 (0.3)

a %CVb, percent coefficient of variance between subjects.

b *R_o_* was calculated as day 14 AUC_0–τ_/day 1 AUC_0–_*_t_*, where *t* and τ equal 24 h.

c *R_o_C*_max_ was calculated as day 14 *C*_max_/day 1 *C*_max_.

The results for AUC to the time of the last quantifiable concentration (AUC_0–_*_t_*) (slope of 1.32) indicated that GSK3036656 increased in a greater-than-dose-proportional manner with increasing dose, which could be attributed partly to extended PK sampling for higher doses. In part B, GSK3036656 appeared to increase in a slightly greater-than-dose-proportional manner for AUC_0–τ_ on day 1 (slope of 1.24); however, by day 14, the increase in AUC_0–τ_ was dose proportional for the 5- to 15-mg doses (slope of 1.06). The increase in *C*_max_ for the 5-mg to 15-mg dose range was dose proportional on day 1 and on day 14 (slopes of 1.19 and 1.06, respectively). The coadministration of food with 5 mg GSK3036656 did not alter the PK of GSK3036656 (Table 5). In addition, there was no clinically relevant difference in time needed to reach the maximum concentration of drug in serum (*T*_max_) following administration of GSK3036656 with food. GSK3036656 reached steady state by day 14 of daily administration of 5 mg and 15 mg. In part B, both doses showed accumulation (Table 6) (2.36- to 2.87-fold for AUC_0–τ_ and 1.73- to 2.01-fold for *C*_max_) of GSK3036656 on day 14 compared with day 1.

**TABLE 5:** Effect of food on plasma pharmacokinetic parameters of GSK3036656^*a*^

Parameter	Treatment (mg)	Comparison	Test		Ref		Ratio	90% CI
			No. of subjects	Geometric LS mean	No. of subjects	Geometric LS mean		
AUC_0–∞_ (h · ng/ml)	5	Fed vs fasted	5	1,348.60	6	1,428.33	0.94	0.71, 1.26
AUC_0–_*_t_* (h · ng/ml)	5	Fed vs fasted	5	786.54	6	731.74	1.07	0.87, 1.34
*C*_max_ (ng/ml)	5	Fed vs fasted	5	46.98	6	48.42	0.97	0.76, 1.23
*t*_1/2_ (h)	5	Fed vs fasted	5	34.35	6	38.64	0.89	0.64, 1.23
*T*_max_ (h)	5	Fed vs fasted	5	2.00	6	1.25	0.75^*b*^	−0.50, 2.50

a CI, confidence interval; LS, least squares; Ref, reference.

b Difference.

**TABLE 6:** Plasma GSK3036656 pharmacokinetic parameters assessing accumulation^*a*^

Parameter	Treatment regimen	Comparison	Test		Ref		Ratio	90% CI
			No. of subjects	Geometric LS mean	No. of subjects	Geometric LS mean		
AUC(*R_o_*) (h · ng/ml)	Repeat 5 mg	Day 14 vs day 1	7	1,392.22	7	485.56	2.87	2.56, 3.21
	Repeat 15 mg	Day 14 vs day 1	8	4,461.26	8	1,891.04	2.36	2.12, 2.62

*R_o_C*_max_ (ng/ml)	Repeat 5 mg	Day 14 vs day 1	7	97.04	7	48.40	2.01	1.69, 2.37
	Repeat 15 mg	Day 14 vs day 1	8	309.55	8	178.79	1.73	1.48, 2.03

a CI, confidence interval; LS, least squares; Ref, reference.

Metabolite profiling studies using pooled plasma and urine revealed that GSK3036656 recovery from spiked plasma was 71% using ultraperformance liquid chromatography (UPLC)-mass spectrometry (MS) analysis. The limit of detection of GSK3036656 was 5 ng/ml, equivalent to 5% and 3% of the derived GSK3036656 plasma concentrations for the single- and repeat-dose pools, respectively. For urine, the limit of detection by UPLC-MS for GSK3036656 was 50 ng/ml, equivalent to approximately 2% of the GSK3036656-related material detected. Overall recovery of GSK3036656 from spiked plasma after the entire preparative high-performance liquid chromatography (HPLC) process was determined to be 44% using nuclear magnetic resonance (NMR) analysis. GSK3036656 could not be isolated from spiked urine, with loss of GSK3036656 assumed to be due to degradation or nonspecific binding. Repeat analysis of pooled and spiked urine suggested that GSK3036656 degraded to GSK3635633 following multiple freeze-thaw cycles and prolonged exposure to light.

After both single and repeat dosing, unchanged GSK3036656 was the only drug-related component detected in plasma. Unchanged GSK3036656 and GSK3635633 (compound M1) were detected in urine, accounting for approximately 90% and 10% of the observed drug-related material, respectively (Tables 7 and 8). The presence of M1 may be attributed to metabolism and/or degradation of GSK3036656; consequently, we believe that the reported amount of GSK3036656 observed in urine may be an underestimate. Based on total drug-related material detected in urine, the minimum absorbed doses after single (25-mg) and repeat (15-mg) dosing were 50 and 78%, respectively. Unchanged GSK3036656 represented at least 44% and 71% of the 25- and 15-mg doses, respectively. Urinary elimination appeared to be protracted, with only 30% of the dose observed in a 0- to 24-h sample collected after a single 15-mg dose, compared to 78% after repeat dosing.

**TABLE 7:** Metabolites identified in pooled plasma following oral single-dose (25 mg) and repeat-dose (15 mg/day for 14 days) administration of GSK3036656^*a*^

Metabolite	Proposed structure	% oDRM, single dose (25 mg), day 1, 0–24 h	% oDRM, repeat dose (15 mg), day 14, 0–24 h
P (GSK3036656)	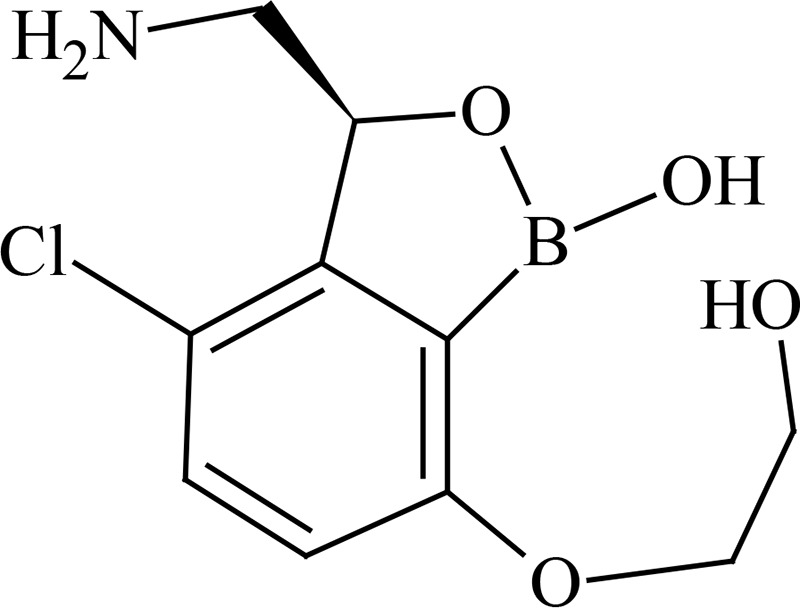	100	100
M1 (GSK3635633), oxidation and deboronation	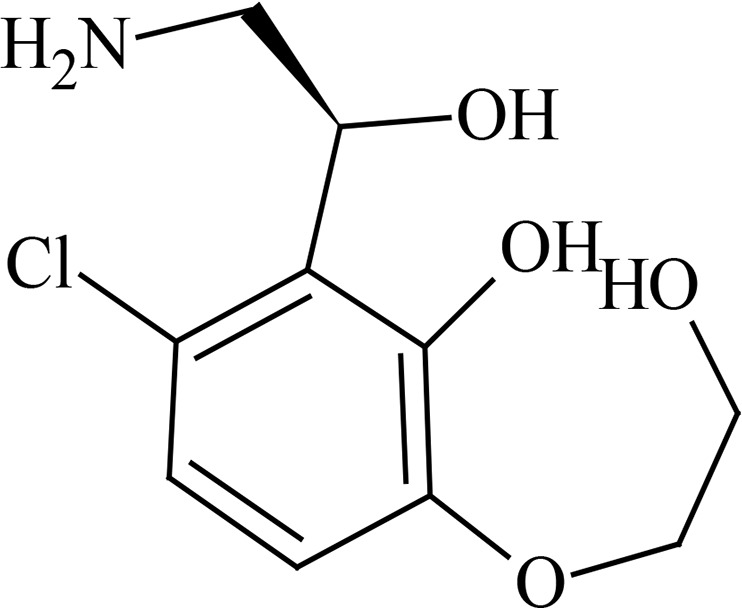	ND	ND

a oDRM, observed drug-related material; ND, not detected.

**TABLE 8:** Metabolites identified in pooled urine following oral single-dose (25 mg) and repeat-dose (15 mg/day for 14 days) administration of GSK3036656^*a*^

Metabolite	Proposed structure	% oDRM, single dose (25 mg), day 1, 0–72 h (% dose)	% oDRM, repeat dose (15 mg), day 1, 0–24 h (% dose)	% oDRM, repeat dose (15 mg), day 14, 0–24 h (% dose)
P (GSK3036656)	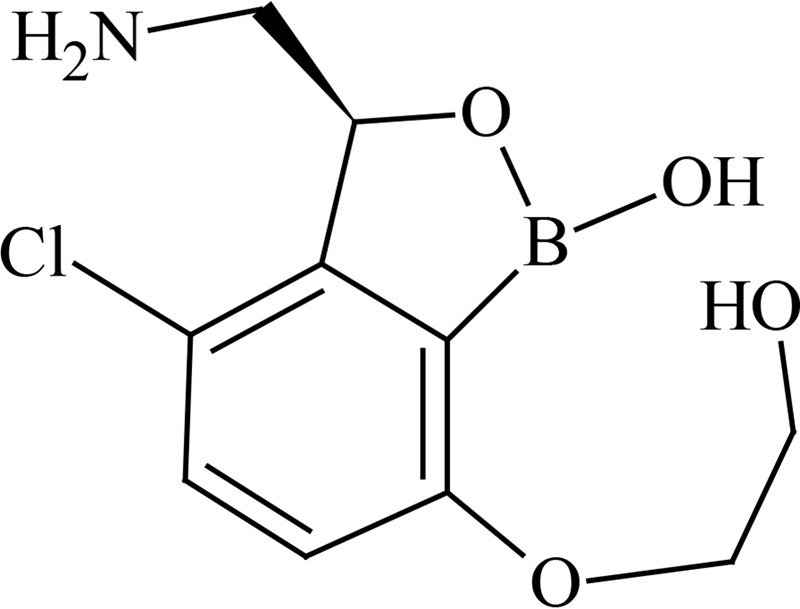	88 (44)	92 (27)	91 (71)
M1 (GSK3635633), oxidation and deboronation	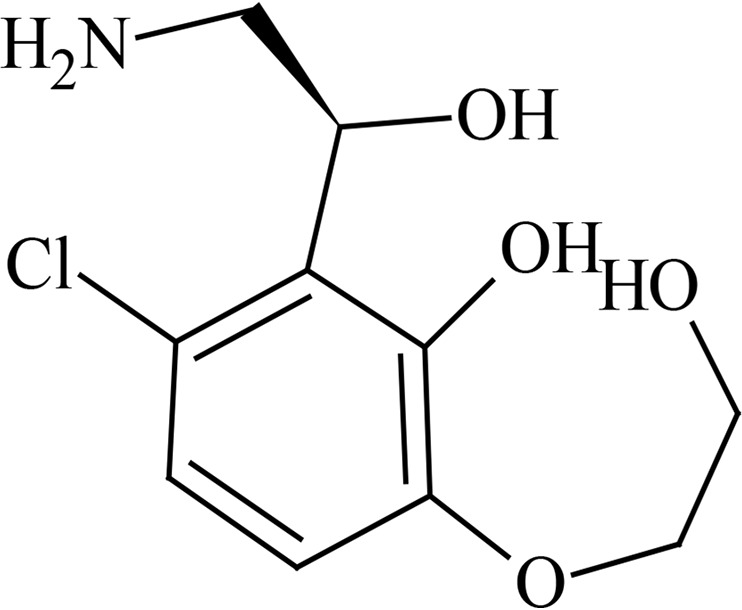	12 (6)	8 (2)	9 (7)

Total % dose		50	30	78

a oDRM, observed drug-related material.

### Population pharmacokinetic modeling and simulations.

Fit-for-purpose population PK models were developed with available concentration-time data, and clinical trial simulations (CTS) were performed to inform dose escalation decisions (15). Proposed doses for escalation were considered acceptable if the predicted probability of an individual within a trial exceeding the exposure limits was ≤10% (Table 9). Beginning with the first analysis (*n* = 6), concentration-time data were adequately described by a 2-compartment model with first-order absorption. Parameters were generally well defined, with standard errors (SE) typically being less than 30%, with increasing precision as the data size increased. Interindividual variability was estimated on up to four parameters (oral clearance [CL/*F*], central and peripheral volumes of distribution [*V*_c_/*F* and *V*_p_/*F*, respectively], and absorption rate constant [*K_a_*]) and was typically <30%, except for *K_a_* (77 to 101%). Visual predictive checks (VPCs) demonstrated adequate model performance (data not shown). An exposure limit was exceeded only in the 15-mg repeat-dosing cohort, where the AUC limit was exceeded in 3 of 8 subjects (a maximum 20% increase above the limit). A fit-for-purpose model was not used for this dose escalation step based on the model using single-dose data alone only slightly underpredicting median steady-state exposure for the 5-mg repeat-dose level.

**TABLE 9:** Clinical trial simulation results and dose escalation decisions

Dose (mg)	% probability of any subject exceeding exposure threshold in a clinical trial	
	AUC_0–24_	*C*_max_
Dose escalation analysis 1^*a*^		
15	0	0
25	0	0
30	0	7
35	4	23

Dose escalation analysis 2^*b*^		
25	0	5.3
30	3.3	20.3

Dose escalation analysis 3^*c*^		
25	0.1	5.8
30	1.5	18

Dose escalation for repeat dose^*d*^		
5	0	0
10	0	0
15	10	1.4

a Data include a 5-mg single dose (*n = 6*) and simulation of 100 trials (*n* = 6/trial), with a decision for a 15-mg single dose.

b Data include 5- and 15-mg single doses (*n* = 12) and simulation of 1,000 trials (*n* = 6/trial), with a decision for a 25-mg single dose.

c Data include 5-, 15-, and 25-mg single doses (*n* = 18) and simulation of 1,000 trials (*n* = 6/trial), with a decision to end the single dose.

d Data include 5-, 15-, and 25-mg single doses (*n* = 18) and simulation (steady state) of 1,000 trials (*n* = 8/trial), with a decision to initiate with 5 mg once daily.

### Predictions of the early bactericidal activity of GSK3036656 in tuberculosis patients.

**FIG 3:**
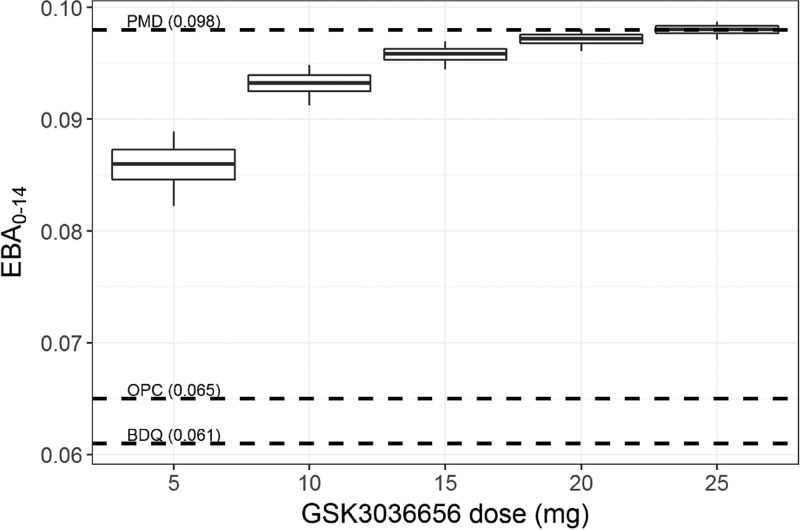
Predicted early bactericidal activity of GSK3036656 in tuberculosis patients. Analysis predicted the early bactericidal activity (EBA_0–14_) following 14 days of treatment with 5 to 25 mg once daily, based on the estimated PK/PD relationship in mouse infection models. Box plots represent the 5th, 25th, 50th, 75th, and 95th percentiles of the simulated individual EBA_0–14_. Numbers in parentheses represent the reported average EBA_0–14_ of each reference drug at therapeutic doses. PMD, pretomanid; BDQ, bedaquiline; OPC, delamanid.

At the completion of the study, available concentration-time data were used to develop a preliminary population PK model. To facilitate the translation of treatment effect from mice to patients, this PK model and a two-state bacterial growth dynamics model (25) were used for CTS (parameters listed in Table 10) to predict the GSK3036656 dose range that produces the highest possible early bactericidal activity (EBA_0–14_) (taking into account the predefined exposure limit). The predicted median EBA_0–14_ ranged from 0.086 (90% confidence interval [CI], 0.082 to 0.089) for 5-mg dosing to 0.093 (90% CI, 0.091 to 0.095) for 10-mg dosing or approximately 0.1 for 15- to 25-mg dosing (Fig. 3). These results suggested 10 to 15 mg as the optimal GSK3036656 dose for TB treatment.

**TABLE 10:** Population PK and PD parameter estimates for clinical trial simulations^*a*^

Parameter	Final model value		
	Estimate (%SE)	Bootstrap (1,000 runs)	
		Median estimate	95% CI
Fixed effects			
Pharmacokinetics			
CL/*F* (liters/h)	3.69 (3)	3.7	3.48–3.91
*V*_2_/*F* (liters)	14.9 (30)	15.9	9.77–24.8
*Q*/*F* (liters/h)	63 (15)	63.1	47.7–81.8
*V*_3_/*F* (liters)	150 (6)	149	132–163
Absorption lag time (h)	0.235 (3)	0.24	0.22–0.24
*K_a_* (h^−1^)	0.925 (23)	0.95	0.65–1.37
Bacterial growth dynamics^*b*^			
*k*_netF_ (h^−1^)	0.0272 (FIX)		
*k*_netS_ (h^−1^)	0.00068 (FIX)		
*B*_max_ (log_10_ CFU/sputum)	6.2 (FIX)		
Inoculum in patient (log_10_ CFU/sputum)	2 (FIX)		
Drug effects			
*E*_max_-F (h^−1^)	0.052 (FIX)^*b*^		
*E*_max_-S (h^−1^)	0.0037 (FIX)^*b*^		
EC_50_-F (mg/liter)	0.0147 (FIX)^*c*^		
EC_50_-S (mg/liter)	0.03 (FIX)^*c*^		

Random effects			
IIV^1^ CL/*F*	16.1 (22)	15.8	11.8–20
IIV^1^ *V*_2_/*F*	111 (37)	107	70.7–140
IIV^1^ *Q*/*F*	27.3 (37)	26.5	17.3–40
IIV^1^ *V*_3_/*F*	23.3 (30)	22.9	17.3–31.6
Residual variability (%) (Prop)	12.9 (16)	12.6	10–14.1

a Based on available data (no covariates were tested). *k*_netF_, net growth rate of a fast-growing population; *k*_netS_, net growth rate of a slow-growing population. IIV, interindividual variability; Prop, proportionality; FIX, fixed parameter.

b The SE for the inoculum and *B*_max_ (bacterial carrying capacity in sputum samples) could not be calculated as these are fixed parameters. SE for net growth rates were <1%.

c Fixed to estimated values from acute and chronic mouse infection models. The SE for *E*_max_-F (maximum killing rate against a fast-growing population) and *E*_max_-S (maximum killing rate against a slow-growing population), were 2% and 5%, respectively, calculated as EC_50,mouse_ × (plasma unbound fraction_656,mouse_/plasma unbound fraction_656,human_), with the EC_50_-F (potency [50% effective concentration] against a fast-growing population) and EC_50_-S (potency against a slow-growing population) in mouse being 0.0156 mg/liter (SE = 13%) and 0.0317 mg/liter (SE = 23%), respectively. Unbound fractions of GSK3036656 in mouse and humans were experimentally determined to be 0.792 and 0.838, respectively.

## DISCUSSION

GSK3036656 is a novel 3-aminomethyl 4-halogen benzoxaborole and is an M. tuberculosis enzyme LeuRS inhibitor being developed for the treatment of TB. A boron atom is an integral part of these M. tuberculosis LeuRS inhibitors, as it forms a bidentate covalent adduct with the terminal nucleotide of tRNA, Ade76. This covalent adduct traps the 3′ end of the tRNA^Leu^ in the editing site forming a nonproductive complex, blocking leucylation and bacterial protein synthesis (17). The amino group of the (*S*)-aminomethyl side chain at C-3 plays an important role in binding, as it leads to three hydrogen-bonding interactions. Also, the presence of a halogen atom (Cl or Br) significantly improves the M. tuberculosis LeuRS activity and antitubercular action against M. tuberculosis H37Rv and is selective against other bacteria.

This was a first-time-in-human study evaluating the safety, tolerability, PK, and food effect after single and repeat doses of GSK3036656. GSK3036656 was generally well tolerated. No SAEs were reported. The most frequently reported AEs in the study were headache (part A and part B, 2 subjects each) and abdominal pain (part A, 2 subjects). Clinically significant changes in alanine aminotransferase (ALT) and aspartate aminotransferase (AST) levels were observed in 1 subject in part B during treatment with 5-mg repeat daily dosing. The changes were attributed to acute infectious mononucleosis, an AE not considered drug related. There were no safety concerns related to clinical laboratory, vital signs, ECG, or telemetry findings.

Plasma PK of GSK3036656 showed a dose-proportional increase following single-dose administration of 5 mg, 15 mg, and 25 mg GSK3036656. Analysis of the *C*_max_ and AUC parameters showed that the multiple-dose regimens of GSK3036656 administered once a day at 5 and 15 mg were dose proportional after dosing for 14 days. Statistical analysis of *C*_max_ and AUC_0–τ_ showed accumulation of GSK3036656 with repeated administration (1.7- to 2.0-fold for *C*_max_ and 2.4- to 2.9-fold for AUC_0–τ_). Steady-state concentrations of GSK3036656 were achieved following 14 daily doses of GSK3036656. Pharmacokinetic parameter values were not altered in the presence of food at the 5-mg dose level. The steady-state AUC after dosing of 15 mg daily (on average, 4,460 ng · h/ml) was approximately 2.5-fold higher than the AUC considered to be a target exposure for efficacy in humans (1,740 ng · h/ml). Pharmacokinetic differences (e.g., in clearance) between healthy volunteers and patients with TB will need to be considered when defining the therapeutic dose.

Preliminary characterization of metabolites of GSK3036656 in human plasma studies revealed that unchanged parent GSK3036656 was the only drug-related component detected in pooled plasma extracts from single- and repeat-dose cohorts. Based on total drug-related material detected in urine, the minimum absorbed doses after single (25 mg) and repeat (15 mg) dosing were 50 and 78%, respectively. GSK3036656 was cleared predominantly unchanged, representing approximately 90% of the observed drug-related material in urine. A single component formed via oxidation and deboronation, GSK3635633 (compound M1) was also quantifiable, accounting for up to 10% of the observed drug-related material in the urine. Urinary elimination was protracted with 30% of the dose observed in a 0- to 24-h sample collected after a single 15-mg dose, compared to 78% after repeat dosing. Analysis of pooled and spiked urine samples suggested that GSK3036656 degrades to GSK3635633 following repeat freeze-thaw cycles and prolonged exposure to light, and therefore, the presence of M1 may be attributed to metabolism and/or degradation of GSK3036656; consequently, the reported amount of GSK3036656 observed in urine may be an underestimate.

CTS, by incorporating interindividual variability with fit-for-purpose PK models, allowed for safe dose escalation during an FTIH study with restrictive exposure thresholds. CTS demonstrated that *C*_max_ was the dose escalation limiting parameter for a single dose and that AUC was the one for a repeat dose. Confidence in the predicted probabilities increased with accumulating data, primarily via the bootstrap procedure employed to provide uncertainty estimates in the simulations. The effect of high interindividual variability and low precision for parameters when modeling small data sets needs to be considered; this may lead to inflation of variability and overconservative predictive probability estimates.

In contrast to previous cases in early drug development for anti-TB compounds, this program ensured that a strictly quantitative approach and integration of preclinical PK/pharmacodynamic (PD) data were utilized to provide a robust dose rationale for the next phase II study. Clinical trial simulations suggest that 10 to 15 mg should be considered the potential optimal dose for GSK3036656. The EBA_0–14_ corresponding to this dose range in drug-susceptible TB patients (∼0.1) is predicted to be of a similar magnitude as the EBA_0–14_ of pretomanid (mean = 0.098) (18) and superior to those of bedaquiline (mean = 0.061) (15) and delamanid (mean = 0.065) (18). Data from the proof-of-concept study will be used to explore differences in PK between healthy subjects and TB patients and to assess the predictive performance of the PK/PD model.

In conclusion, this FTIH study demonstrated that GSK3036656 was safe and generally well tolerated after single and multiple doses. The PK properties and metabolite profiling exhibited by GSK3036656 make it a promising antitubercular agent for further clinical development with a novel mechanism of action and with a shorter duration of treatment.

## MATERIALS AND METHODS

### Study design and subjects.

This was a randomized, double-blind, placebo-controlled FTIH study (ClinicalTrials.gov identifier NCT03075410). The primary objectives of the study were to investigate the safety, tolerability, and PK of GSK3036656 after single ascending and repeat oral doses in healthy adult subjects. The secondary objectives of the study were to (i) assess the effect of food on the PK properties of GSK3036656 following a single oral dose, (ii) assess preliminary dose-exposure proportionality of GSK3036656 following single and repeat oral doses, (iii) examine the extent of accumulation and achievement of steady state following repeat oral doses of GSK3036656, and (iv) define the metabolic profile of GSK3036656 by identifying and quantifying GSK3036656-related material in plasma and urine.

The study enrolled healthy male and female subjects aged between 18 and 55 years, with a body weight of ≥60 kg, a body mass index (BMI) of between 19 and 29.9 kg/m^2^ inclusive, and no clinically significant abnormalities in vital signs or electrocardiographs (ECGs) or in chemistry, hematology, or liver function tests. Key exclusion criteria included women of childbearing potential; pregnant or lactating females; and individuals with current or chronic history of liver disease or known hepatic or biliary abnormalities, the presence of a moderate or severe cardiac valve disorder, alanine aminotransferase (ALT) and bilirubin levels >1.5× the upper limit of normal (ULN), an ECG QT interval corrected for heart rate using Fridericia’s formula (QTcF) of >450 ms, concomitant use of prescription or nonprescription drugs, history of smoking or heavy alcohol use, and positive HIV or hepatitis B or C virus status. All subjects gave written informed consent. The protocol and informed-consent form were approved by the Office for Research Ethics Committees Northern Ireland (ORECNI), United Kingdom.

Safety was assessed during the conduct of the study by physical examination (height, weight, and cardiovascular, respiratory, neurological, and gastrointestinal systems), monitoring of vital signs (blood pressure, heart rate, tympanic temperature, and respiratory rate, etc.), 24-h Holter monitoring, and recording of ECGs at predefined time points. Clinical chemistry, hematology, and urine analysis were done on day 1, and at 24 h and 72 h postdose during the single-dosing period and for repeat dosing on day 1, and in the morning (predose if feasible) on days 4, 6, 10, and 15. All adverse events (AEs) and serious adverse events (SAEs) experienced during the conduct of the study were reported and assessed for their intensity and causality. Follow-up of AEs and SAEs was done according to the protocol. A detailed record of concomitant medication was maintained. Telemetry was recorded from −1 h predose until 24 h postdose on day 1. ECGs were recorded at predosing and at 10 different time points after dosing on day 1 during single dosing and at multiple time points on day 1 and day 14 during repeat dosing (predose on days 6 and 10), and one ECG was recorded at the follow-up of the study. Triplicate measurements were taken predose (2 to 5 min apart), and single measurements were taken thereafter. In light of the nonclinical safety findings, specific cardiovascular safety monitoring was implemented using cardiac troponin assessment and echocardiograms at baseline and follow-up.

The study had two parts, part A (single ascending dose), employing a crossover design, and part B (repeat dose). A 14-day washout period between each dose level was planned, based on an ∼30-h predicted half-life of GSK3036656. In part A, one cohort of 9 subjects (*n* = 6 for GSK3036656 and *n* = 3 for placebo) was recruited, with subjects taking part in each of 4 treatment periods of 5 mg, 15 mg, 25 mg, and 5 mg (fed). The starting GSK3036656 dose was 5 mg. As this was an FTIH study, each treatment (dosing) period was commenced with a sentinel group of 2 subjects. On day 1 of each dose level, 1 subject received GSK3036656, and 1 subject received placebo (2 sentinel subjects). The remaining subjects within the dosing group were dosed 1 day later, provided that satisfactory safety and tolerability were demonstrated for the 2 sentinel subjects dosed on day 1. Postdose, subjects went for safety and PK assessments for at least 72 h. In part B, up to 4 sequential cohorts were planned to be recruited to repeat doses of 5 or 15 mg of GSK3036656 or placebo once daily for 14 days, with 10 subjects per cohort (*n* = 8 for GSK3036656 and *n* = 2 for placebo per dose level). Postdose, subjects were assessed for PK profiles (until 72 h) on day 1 and day 14, and predose, subjects were assessed on days 12 and 13. A follow-up 14 days after completion of the last dose was performed in both parts. Total durations of the study for each subject in parts A and B were 12 and 8 weeks, respectively. Except for paracetamol, other drugs were contraindicated during the study duration. The dosing of a cohort was agreed upon by the dose escalation committee following a review of the safety data from sentinel dose administration. Appropriate doses and dose regimens for part B were selected by the dose escalation committee based on available safety, tolerability, and PK data from part A and/or any preceding repeat-dose cohorts from part B. The decision to move forward was based on the review of safety data from at least the first week of dosing and PK data from at least the first 48 h of dosing at the preceding dose.

### Food effect study.

An assessment of the effect of food on the exposure to GSK3036656 was incorporated into the single-dose part of the study (part A). This assessment was done by administering a single dose of GSK3036656/placebo with a high-fat meal provided that this same dose was well tolerated in a previous treatment (dosing) period in the same cohort when administered in the fasted state. The 5-mg dose to be administered with food was selected such that when administered in the fasted state, the predicted exposure was no higher than 50% of the highest exposure allowed in the study, in the eventuality that the presence of food unexpectedly enhanced absorption.

### Plasma and urine samples.

Blood samples for plasma drug concentration quantification analysis were collected at predefined time points, which for part A included predose and 0.25, 0.5, 0.75, 1, 1.5, 2, 3, 4, 6, 8, 12, 15, 24, 36, 48, and 72 h after dosing on day 1, with the sampling time extended to 144 h based on emerging clinical data. For part B, blood samples were collected at the same time points as the ones listed above for part A and on days 1 and 14, including a predose sample on days 12 and 13. Blood samples (2 ml) were collected via an indwelling cannula (or vein puncture) into anticoagulant K_2_EDTA tubes and immediately placed on ice. At the 15- and 24-h time points, 5-ml blood samples were collected. Two plasma aliquots were made, one for PK analysis (0.5 ml) and the remainder for metabolite analysis. Plasma was separated by centrifugation at 1,500 × *g* at 4°C for 10 min and then stored at −20°C or below prior to analysis. Urine samples were collected from each subject into preweighed polypropylene containers at predefined time points, which for part A included for predose and 0 to 24, 24 to 48, and 48 to 72 h after dosing on day 1. For part B, samples were obtained predose and 0 to 24 h after dosing on days 1 and 14. At the end of each urine collection period, weight was recorded, volume was calculated based upon weight divided by 1.02 (specific gravity of urine), and an aliquot was transferred to a plastic container and stored at −20°C before analysis.

### Bioanalytical methods for determination of GSK3036656 concentrations and metabolite profile in plasma and urine.

Robust and reproducible bioanalytical methods were developed, validated, and used for determination of plasma concentrations and the metabolite profile of GSK3036656 in plasma and urine. Plasma samples were analyzed using a validated protein precipitation analytical method followed by high-performance liquid chromatography (HPLC-tandem mass spectrometry (MS/MS) analysis of GSK3036656 by IVIVT Bioanalysis, GlaxoSmithKline, Ware Hertfordshire, United Kingdom. GSK3036656 was extracted from human plasma by protein precipitation using acetonitrile containing [^2^H_6_^11^B^13^C]-GSK2982434 as an internal standard. Extracts were analyzed by HPLC-MS/MS using a TurboIonSpray interface with positive-ion multiple-reaction monitoring. This method was validated over the range of 10 to 10,000 ng/ml, and the lower limit of quantification (LLQ) was 10 ng/ml using a 50-μl aliquot of human plasma. Quality control (QC) samples, containing GSK3036656 at 3 different concentrations (30, 400, and 8,000 ng/ml) stored with study samples, were analyzed with each batch of samples against separately prepared calibration standards. For the analysis to be acceptable, no more than one-third of the QC results were to deviate from the nominal concentration by more than 15%, and at least 50% of the results from each QC concentration should be within 15% of nominal.

Analysis to obtain preliminary structural information and quantitative estimates on the metabolites of GSK3036656 in human plasma and urine was performed on the samples obtained from single-dose (25 mg), repeat-dose (15 mg/day for 14 days), and placebo dose groups. Human plasma from 6 subjects within each dose group was pooled, representative of the AUC over the range of 0 to 24 h, using a method described previously by Hop et al. (17). A urine pool (0 to 72 h) was prepared for the single-dose subjects (*n* = 6; 0.09% of the total volume), and separate urine pools (0 to 24 h) were prepared for repeat-dose subjects by combining proportional volumes (*n* = 8; 0.12% and 0.10% of the total volume for days 1 and 14, respectively). Placebo group samples were made by combining equal volumes of predose urine from single-dose (*n* = 6; 2 ml) and repeat-dose (*n* = 8; 4 ml) groups.

Plasma pretreatment was done by extracting 0.3 ml of plasma with 0.9 ml of 90% acetonitrile–10% ethanol, followed by centrifugation at 5,000 × *g* at room temperature for 10 min. Supernatants were evaporated under nitrogen at 25°C to dryness, reconstituted with water (0.3 ml), and vortexed before UPLC-MS analysis. Control plasma extracts were prepared in a similar fashion. In addition, pooled plasma samples were extracted by vortex mixing single- and repeat-dose aliquots (15 ml and 20 ml) with 45 ml and 60 ml of 90% acetonitrile–10% ethanol, followed by centrifugation at 1,800 × *g* at room temperature for 10 min. Supernatants were evaporated under nitrogen at 25°C to 10 ml and fractionated by preparative HPLC.

The preparative HPLC conditions set were an Agilent 1260 LC pump, an Agilent 1260 autosampler, and a UV detector (wavelengths of 210 and 230 nm). The mobile phase consisted of solvent A (20 mM ammonium acetate in D_2_O [pH 5.5]) (acetic acid) and solvent B (acetonitrile). The column (Xbridge Prep C_18_ [5 μm; 250 mm by 10 mm]) was eluted at a flow rate of 3 ml/min, with an injection volume of 5 ml/min pump on for 2 min. The gradient was set to be 5% solvent B at the start, increasing to 35, 40, and 95% at 37, 42, and 52 min, respectively. HPLC-MS/MS analysis was done using an Agilent 6120 instrument with Chemstation software (v A02.1.2), an Electrospray interface with positive-ion multiple-reaction monitoring. Recovery of GSK3036656 from plasma was performed by extraction of spiked plasma (1,000 ng/ml into 0.2 ml plasma), followed by reconstitution with 0.25 ml water. Analysis was done using UPLC-MS. The limit of detection for GSK3036656 was 5 ng/ml, equivalent to 5% concentrations for the single and repeat doses.

For urine, the limit of detection by UPLC-MS for GSK3036656 was 50 ng/ml, equivalent to approximately 2% observed drug-related material. For the recovery of GSK3036656 from urine after preparative HPLC and NMR (nuclear magnetic resonance) spectroscopy analysis, 0.05 ml of a standard solution of GSK3036656 (1 mg/ml) was spiked into 400 ml of control human urine. Spiked samples were fractionated with and without freeze-drying by preparative HPLC under a variety of conditions, and the resulting fractions were analyzed by ^1^H NMR spectroscopy. In addition, appropriate pooled control plasma extracts and urine were examined to aid in the distinction of drug-related material from endogenous components. The plasma fractions isolated by preparative HPLC and eluted in deuterated solvents were transferred for NMR analysis to provide additional structural information and to estimate the levels of drug-related material present.

### Pharmacokinetics and statistical analyses.

Plasma pharmacokinetic parameters for each subject based on actual sampling time were estimated by noncompartmental analysis using WinNonlin version 6.3 (Pharsight Corporation, Cary, NC, USA). From the concentration-time data, the maximum drug concentration in plasma (*C*_max_); time needed to reach the maximum concentration (*T*_max_); areas under the concentration-time curve to the time of the last quantifiable concentration, extrapolated to infinity, and over the dose interval (AUC_0–_*_t_*, AUC_0–∞_, and AUC_0–τ_, respectively); elimination half-life (*t*_1/2_); and trough concentrations (*C*_τ_) were calculated. The linear-up log-down trapezoidal method was used for calculating AUC, and at least 3 data points in the terminal phase were used in the determination of AUC_0–∞_ and *t*_1/2_. Pharmacokinetic data were descriptively summarized. *C*_τ_ values collected on the specified days were used to assess attainment of steady state. To estimate the extent of accumulation after repeat dosing, the observed accumulation ratio (*R_o_* [day 14 AUC_0–τ_/day 1 AUC_0–t_] and *R_o_C*_max_ [day 14 *C*_max_/day 1 *C*_max_]) was determined. Dose proportionality was assessed following single doses (part A) via analyses of AUC_0–_*_t_*, AUC_0–∞_, and *C*_max_ and following repeated dosing (part B) using AUC_0–τ_ and *C*_max_.

A mixed-effect model (SAS software, version 9.4, of the SAS System for Unix; SAS Institute Inc., Cary, NC, USA) was used to assess food effect for the log-transformed parameters AUC_0–_*_t_*, AUC_0–∞_, and *C*_max_. Treatment in the fed/fasted state was fitted as a fixed effect, and subject was fitted as a random effect. A fixed-effect model was used to assess the food effect for the log-transformed parameter *t*_1/2_; the model included treatment in the fed/fasted state as a fixed effect. Point estimates and corresponding 90% confidence intervals (CIs) were constructed for the comparisons of interest of fed-fasted, using the residual variance. These were then back-transformed to provide point estimates and corresponding 90% CIs for the geometric mean ratios of fed/fasted. *T*_max_ was analyzed nonparametrically using a Wilcoxon rank sum test to compute the point estimate and 90% CI for the median difference (fed-fasted). The extent of accumulation of GSK3036656 was based on AUC(*R_o_*) and *C*_max_ (*R_o_C*_max_). Following log*_e_* transformation, data for AUC_0–τ_ on day 14 and AUC_0–_*_t_* on day 1 were analyzed by a mixed-effect model (where both τ and *t* equal 24 h). The point estimates and associated 90% CIs were then exponentially back-transformed to provide point and 90% CI estimates for the ratios of “day 14/day 1” for each active dose. Attainment of steady state was based on *C*_τ_. A mixed-effect model of the form *C*_τ_ = α + dose + day + dose × day + subject was fitted with dose, day, and dose-by-day interaction as fixed effects (with dose as a categorical covariate and day as a continuous covariate) and with subject as a random effect. The coefficients of the slopes for the day effect for each dose, along with corresponding 90% CIs, were used to determine whether steady state was achieved. For assessment of dose proportionality, a power model of the form *y* = α × dose^β^, where *y* is AUC or *C*_max_ and α is a random subject effect, was performed on log*_e_*-transformed data. Estimates of the mean slopes of dose were reported along with corresponding 90% CIs.

### Population pharmacokinetic modeling and simulations.

Fit-for-purpose population PK models were developed with available concentration-time data utilizing nonlinear mixed-effects modeling (NONMEM v7.3) (19) to support most dose escalation steps (20). Bootstrapping and visual predictive checks (VPCs) (PsN v3.7.6 and R version 3.2 with RStudio v0.99.902) (21–23) were conducted to assess model performance. Clinical trial simulations (CTS) using parameters from the PK models were conducted, incorporating parameter uncertainty and intersubject variability (mrgsolve in R) (16). Proposed doses for escalation were considered acceptable if the predicted probability of an individual within a trial exceeding the predefined exposure limits for AUC and *C*_max_ was ≤10%.

### Predictions of the early bactericidal activity of GSK3036656 in tuberculosis patients.

At the completion of the study, available concentration-time data were used to develop a population PK model (no covariates were available) (24). This PK model was used to simulate plasma concentrations in humans. In addition, the two-state bacterial growth dynamics model was integrated with a pharmacokinetic/pharmacodynamic (PK/PD) model to describe the drug effects and predict early bactericidal activity (EBA_0–14_) for GSK3036656 in TB patients following a 14-day treatment period based on a once-daily dosing regimen. EBA_0–14_ was defined as (log_10_ CFU day 14 − log_10_ CFU day 0)/14 (25). The bacterial growth dynamics model assumed the existence of fast-growing (F) and slow-growing (S) M. tuberculosis populations and was initially developed based on disease progression data (as measured by CFU/lung counts) obtained in mouse TB infection models (26). Details of the full bacterial growth dynamics model building, including the model structure and supporting differential equations, were reported previously (25). Data that were used for bacterial growth dynamics model building were obtained in the literature (27, 28). PK/PD parameters were estimated from GSK3036656 efficacy data obtained in the acute and chronic mouse infection models as described in the introduction and then scaled to humans based on interspecies differences in disease (ratio between F and S M. tuberculosis populations and baseline CFU levels) and drug-specific (protein binding) properties (25). CTS (1,000 iterations) were subsequently performed to predict the GSK3036656 dose range that produces the highest possible EBA_0–14_ with consideration of the predefined exposure limit, taking into account the available preclinical safety and efficacy data. Published average EBA_0–14_ values for pretomanid (200 to 1,200 mg), bedaquiline (loading doses of 700 mg on day 1 and 500 mg on day 2 and then 400 mg from day 3 onward), and delamanid (300 mg) (15, 18, 29) were used for reference purposes but were not part of the dose selection criteria.

### Data availability.

Anonymized individual participant data and study documents can be requested for further research from www.clinicalstudydatarequest.com.
